# Building bridges across disciplines: Perspectives on interdisciplinary coastal research

**DOI:** 10.1017/cft.2026.10049

**Published:** 2026-09-09

**Authors:** Caroline Hallin, Peter Ruggiero, Sierd de Vries, Åsa Knaggård

**Affiliations:** 1 Department of Building and Environmental Technology, Lund University, Sweden; 2 College of Earth, Ocean and Atmospheric Sciences, Oregon State University, Corvallis, OR, USA; 3 Department of Hydraulic Engineering, Delft University of Technology, Delft, Netherlands; 4 Department of Political Science, Lund University, Sweden

**Keywords:** interdisciplinary research, research collaboration, coastal adaptation, climate change, coastal engineering

## Abstract

Coastal communities face increasing risks from sea level rise, erosion, flooding and growing pressure on coastal land and resources. Addressing these challenges requires research that connects technical, environmental, social, creative and governance outlooks. In this perspective article, we reflect on how interdisciplinary coastal research can be achieved and better supported, drawing on selected literature and our own experiences from European and US coastal engineering research contexts. We argue that meaningful interdisciplinary collaboration depends on both profound disciplinary expertise and reciprocal learning across disciplinary boundaries. To support this discussion, we introduce two new concepts. *The hook-shaped researcher* illustrates how researchers from different disciplines can jointly develop an interdisciplinary research space through partial but mutual knowledge exchange. *The interdisciplinary bridge model* highlights the need for meaningful, challenging and relevant collaboration across all disciplines involved. We identify time, trust, strong relationships, distributed leadership and supportive institutional structures as central conditions for successful interdisciplinary work. We also discuss how funding schemes, publication practices and academic reward systems can better recognize and enable interdisciplinary collaboration. Strengthening these conditions can improve coastal adaptation research and support sustainable responses to complex coastal challenges.

## Impact statement

Coastal communities around the world are facing growing risks from sea-level rise, erosion, flooding and increasing pressure on coastal land and resources. Understanding complex coastal problems and developing sustainable solutions requires collaboration across disciplinary boundaries. In this perspective article, we contribute to that effort by offering practical reflections on how interdisciplinary coastal research can be supported and strengthened. We introduce two concepts, the hook-shaped researcher and the interdisciplinary bridge model, that can help researchers and institutions reflect on what makes collaboration across disciplines meaningful, productive and sustainable. These concepts highlight the importance of deep disciplinary knowledge, reciprocal learning and strong relationships between researchers in achieving high-level interdisciplinary research. The wider benefit of this work is that it provides language and models that can be used by research teams, universities, funders and journals to better design, evaluate and support interdisciplinary coastal research. By identifying conditions that support collaboration, this perspective can help improve how coastal adaptation research is organized and how researchers are trained, funded and rewarded. Ultimately, stronger interdisciplinary collaboration can lead to coastal research that is better equipped to address real-world climate adaptation challenges and to support sustainable development of coastal areas.

## Introduction

In 1988, Dean et al. (1988) concluded that 80–85% of erosion on the east coast of Florida was caused by human activities related to stabilizing coastal inlets. It is a classic example of solving one problem while creating another. When inlets were stabilized for navigational purposes, downdrift erosion began, triggering a cascading chain of coastal protection and adaptation measures with associated economic and environmental impacts (Houston and Dean, 2016).

Four decades later, coastal engineers find themselves operating in a world increasingly impacted by sea level rise (SLR), changing patterns of storminess and the uncertainties associated with future climate change (Intergovernmental Panel on Climate Change (IPCC), 2023). Already, current rates of SLR are aggravating multiple coastal risks; SLR is a direct driver of erosion, increased frequency and severity of coastal flooding, saltwater intrusion, groundwater salinization and drainage problems in low-lying areas (Lincke and Hinkel, 2018; van de Wal et al., 2024).

Despite the increasing impact and costs of natural hazards along the coast, population densities in risk-exposed coastal areas continue to increase (Barragán and de Andrés, 2015; Reimann et al., 2023; Rentschler et al., 2023). By 2100, the number of people living in annually flooded coastal areas is estimated at 190 million in low-emission scenarios and up to 630 million in high-emission scenarios (Kulp and Strauss, 2019).

As a consequence, coastal protection measures are increasingly being implemented at larger scales. A recent study estimated that 33% of the world’s sandy coastlines have been hardened by coastal protection (Nawarat et al., 2024), and the transformation of developed coasts from soft to hard is likely to continue with SLR and population growth as the main drivers (Vousdoukas et al., 2020). The hardening is being carried out through incremental changes toward a new coastal landscape, and, as with inlet stabilization on the Florida east coast, it is causing a cascading impact on coastal and marine environments at a global scale (de Vriend et al., 2015; van de Wal et al., 2024).

Meanwhile, the often preferred nature-based solutions (Hanson et al., 2002; Elko et al., 2021; Brand et al., 2022) are facing increasing competition with other activities, for example, conflicts between offshore wind farms and sand nourishment borrow areas (Kusters et al., 2025). Other climate change adaptation strategies, such as managed retreat or relocation, face multiple barriers, including psychological, institutional, and practical ones (Siders, 2019; Hanna et al., 2021; Koslov et al., 2021), and are rarely implemented in coastal areas (Hino et al., 2017; Braamskamp and Penning-Rowsell, 2018).

Climate change adaptation in coastal areas is a ‘wicked’ problem (Rittel and Webber, 1973; Termeer et al., 2016; Head, 2022) characterized by deep uncertainty, contested values and often no definitive solution, which requires expertise from several disciplines and sectors to come together. In response, research and innovation initiatives that cross-cut disciplines and sectors are being scaled up, for example, through funding from the EU Mission on Adaptation to Climate Change and Sea Grant initiatives by the US National Oceanographic and Atmospheric Administration and the Coastlines and People Program of the US National Science Foundation.

Research involving multiple disciplines and sectors is often categorized as multi-, inter- or transdisciplinary (Institute of Medicine, 2005). These terms are typically defined along a continuum, with increasing levels of integration and increasing involvement of nonacademic actors (Aboelela et al., 2007), although definitions vary in the literature (Institute of Medicine, 2005; Stokols et al., 2008).

In this article, we define multidisciplinary research as when two or more disciplines focus on the same problem, topic or theme, fostering broader knowledge while remaining separate. In interdisciplinary research, methods and tools from different disciplines are combined in ways that go beyond the simple sum of the parts. Interdisciplinary research may, for example, involve jointly formulating research questions, transferring a method from one field to another or developing entirely new concepts at the boundaries between the disciplines involved. It is an activity that takes place in the research space between disciplines. Transdisciplinary research goes beyond interdisciplinarity within academia and involves actors from the public and private spheres, emphasizing mutual learning and knowledge integration to address real-world problems (Polk, 2015). In recent years, the concept of convergence has emerged to describe partnerships among actors from different sectors and disciplines to stimulate innovation beyond the scope of transdisciplinary research (National Research Council, 2014; Peek et al., 2020).

Coastal research, especially on climate change adaptation, is often situated within transdisciplinary and convergence contexts (Tintoré et al., 2009; Martinez et al., 2018). However, collaboration among academic researchers and research teams within these broader constellations remains uni-, multi- or interdisciplinary. Each form of collaboration can serve a valuable purpose and generate knowledge and innovation. This article focuses on interdisciplinary research because it requires researchers not only to work alongside one another, but also to develop shared problem framings and integrate disciplinary perspectives. These demands make the intercollegial collaboration especially challenging (MacLeod, 2018).

Previous research identifies recurring barriers to interdisciplinary collaboration, including conflicting terminology and epistemologies, power imbalances, devaluation of others’ contributions and weak leadership (Institute of Medicine, 2005; Eigenbrode et al., 2007; MacMynowski, 2007). These barriers may be reinforced by a lack of recognition of team contributions and inadequate time and funding to establish close working relationships (Institute of Medicine, 2005). They may also become more pronounced as disciplinary distance increases, as often occurs when natural and social sciences are integrated to address wicked societal problems such as coastal adaptation (MacMynowski, 2007; Uhlenbrook and De Jong, 2012; Budtz Pedersen, 2016; Richter et al., 2022).

In this perspective, we reflect on conditions that may support interdisciplinary coastal research and introduce two visual concepts, the hook-shaped researcher and the bridge model, to illustrate these conditions. We draw on selected literature and our personal experiences with coastal engineering research in European and US contexts. The primary audience is coastal researchers, although the discussion may also be relevant to researchers in other fields, as well as to research leaders, universities, funders, journals and professional societies.

## Coastal engineering and interdisciplinary research

Coastal engineering is commonly defined as a branch of civil engineering concerned with the planning, design, construction and maintenance of works in the coastal zone (Bosboom and Stive, 2023). It is a discipline at the interface between nearshore oceanography and coastal geomorphology combined with geotechnical, environmental, structural and hydraulic engineering principles (Sorensen, 2006). Coastal engineers have diverse educational backgrounds and often move between disciplines. Given the nature of coastal engineering projects, coastal engineers also frequently interface with other disciplines and stakeholders throughout the planning, design, implementation, and maintenance of these projects.

There are several examples of successful researchers in the field who have generated new knowledge for coastal engineering by applying methods and models from other fields, such as mathematics (Hulscher et al., 1993) or geophysics (Stark et al., 2022). The societal and environmental contexts of coastal engineering problems are increasingly stimulating collaboration with a wide range of disciplines, for example, with ecologists to design nature-based coastal protection measures that preserve or enhance coastal biodiversity (Möller et al., 2014; Gittman et al., 2016; Narayan et al., 2016), with geologists to develop models for predicting coastal evolution at timescales exceeding the traditional decadal engineering horizon (van der Spek and Beets, 1992; Stolper et al., 2005) and with spatial planners and landscape designers in the context of coastal adaptation (Haasnoot et al., 2013; Berger et al., 2020). In recent years, collaboration with the social sciences and humanities (SSH) has also increased and has been encouraged in EU Horizon projects (Budtz Pedersen, 2016), with the explicit objective to integrate SSH and STEM disciplines (Science, Technology, Engineering and Mathematics) (European Commission, 2025), as well as in US National Science Foundation’s (NSF) Coastlines and People program (Fox et al., 2023; Meselhe et al., 2025).

A recent Decadal Visioning Workshop and paper, led by the US Coastal Research Program, have developed a suite of recommendations for the coastal research community, including embedding social science into coastal research and supporting funding mechanisms that advance interdisciplinary, holistic, community-driven solutions to coastal challenges (Stockdon et al., 2026). The general trend is that inter- and transdisciplinary coastal research is increasing, and that collaboration is extending to disciplines more distant from coastal engineering. As research disciplines become increasingly complex and the body of knowledge expands, it is becoming more difficult to master multiple disciplines at a high level (Jones, 2009). While the era of the polyhistor largely ended in the nineteenth century, it is increasingly important for modern researchers to possess skills for interdisciplinary collaboration.

## Skills for interdisciplinary collaboration

**Figure 1.**
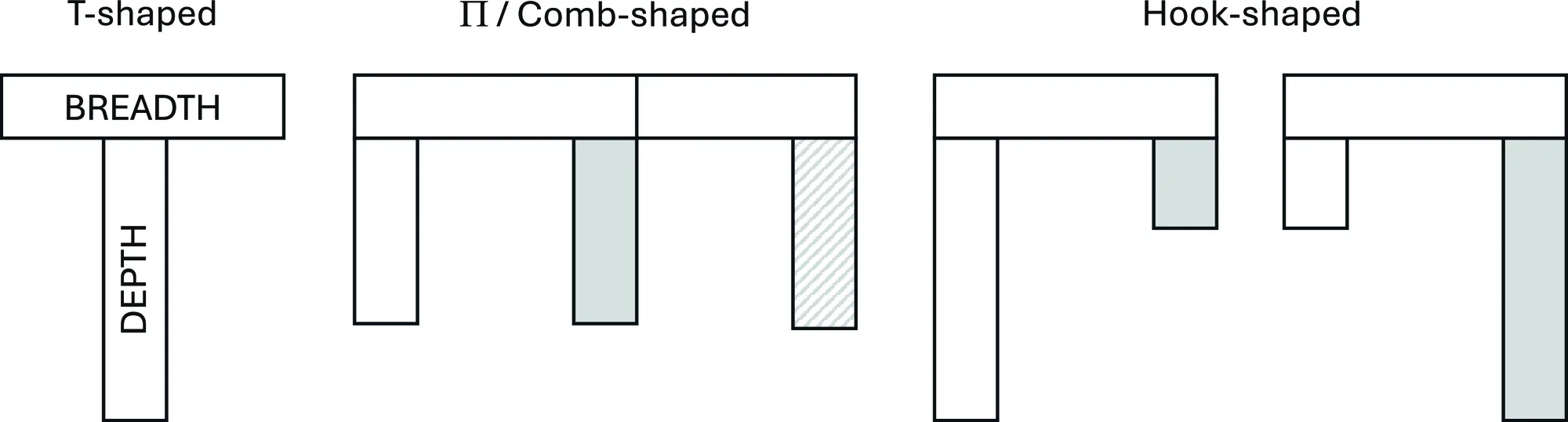
Illustration of the T-shaped, П/comb-shaped and hook-shaped researchers. The vertical scale indicates disciplinary depth in one or several disciplines, while the horizontal scale indicates broad knowledge about the research topic. The hook-shaped model is shown here as two collaborating researchers from different disciplines (white and gray). Collaboration may also occur in the T-shaped and Π/comb-shaped models, but this is not explicitly shown in the figure.

A foundation for interdisciplinary research is profound knowledge of one’s own discipline, combined with a broad and flexible understanding of the wider research problem (Horn et al., 2022). This combination of deep and broad knowledge can be illustrated by the T-shaped researcher (Conley et al., 2017) (Figure 1). The US National Research Council illustrates individuals who master several disciplines as having a П-shaped or comb-shaped profile (National Research Council, 2014, p. 62), sometimes denoted as generalists (Uhlenbrook and De Jong, 2012). However, as discussed in the previous section, achieving individual interdisciplinarity is becoming increasingly difficult.

To illustrate how researchers from different disciplines can jointly develop an interdisciplinary research space through partial but reciprocal knowledge exchange (Barile et al., 2012; Horn et al., 2022), here we propose the hook-shaped researcher model (Figure 1). Unlike the T-shaped model, it emphasizes reciprocal relationships rather than the breadth of a single individual. Learning enough about another discipline can facilitate communication, reveal the limits of one’s own knowledge, and reduce the risk of devaluing others’ contributions (Lélé and Norgaard, 2005).

To develop sufficient reciprocal understanding, researchers need to learn about one another’s disciplines, a development process that is often considered rewarding (Hall et al., 2012; Apine et al., 2026). Communication is facilitated by the ability to adapt language and communication modes, as well as by the ability to view one’s own knowledge from the perspective of other disciplines or epistemologies (Bracken and Oughton, 2006). Since these are skills that are typically developed through pedagogical practice, teaching scientists may be particularly amenable to the hook-shaped model.

There is a widely held view that researchers need to develop a common language and agree on shared concepts and assumptions in order to collaborate effectively across disciplines (Bracken and Oughton, 2006). However, this can be a time-consuming and challenging task (National Research Council, 2014, pp. 64–65), especially for disciplines characterized by fundamental controversies. Rather than striving for agreement on joint formulations, in some situations it is more constructive to just learn about and respectfully accept alternative theories and terminologies (Lélé and Norgaard, 2005; Pohl et al., 2021). ‘Boundary objects’ can support this process (Star and Griesemer, 1989). These are artifacts over which several disciplines can have a shared understanding but within each discipline can have a different and more distinct meaning. They allow researchers to collaborate without necessarily agreeing on every concept or terminology.

Ultimately, researchers must be able to create, enter and operate within an interdisciplinary research space. This requires a degree of pragmatism that can be developed through training in interdisciplinary collaboration. At the same time, such training should not compete with disciplinary formation, since disciplines provide important conceptual and methodological grounding and support the development of professional and scholarly identity.

The optimal educational level for interdisciplinary training may differ depending on future career paths. Students leaving academia may need to develop these skills already at the bachelor’s or master’s level. In contrast, PhD students and postdoctoral researchers may benefit from first developing in-depth disciplinary expertise, while also being challenged to place themselves in situations where they are not experts (Knaggård et al., 2018). In conclusion, the ability to engage in interdisciplinary collaboration can be considered a soft skill that, like academic writing or problem-solving, needs to be trained at all educational levels.

Examples of interdisciplinary training include summer courses, courses in other disciplines, cross-disciplinary conferences, research visits, internships and collaborative teaching. Wingren (2018; 2019), for example, describes an exploratory landscape-architecture course in which Master’s students used walking, dance and choreography to investigate how SLR could transform coastal landscapes. In our experience, collaborative teaching and supervision can provide a good basis for building interdisciplinary research relationships and skills.

## Building interdisciplinary teams

**Figure 2.**
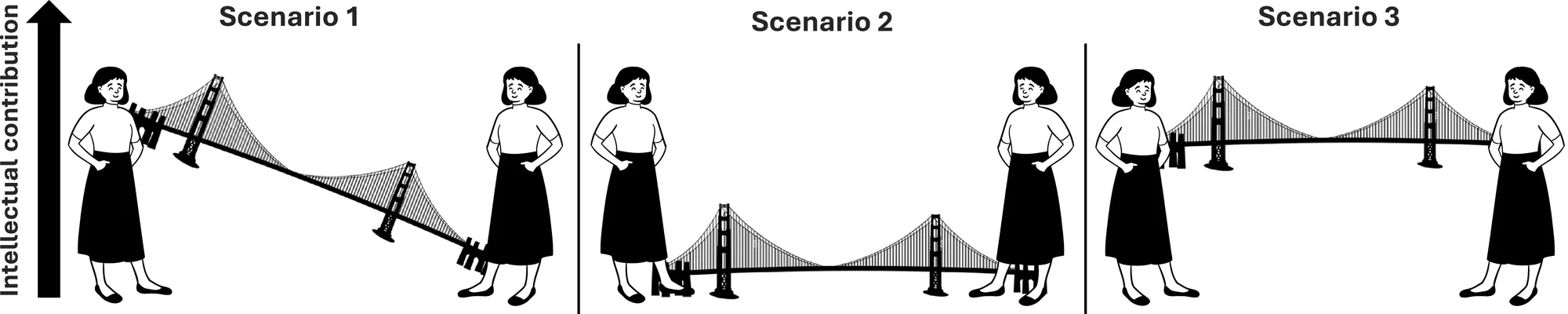
The bridge model, illustrating three scenarios for interdisciplinary collaboration with varying levels of intellectual contribution from each participating research field. Figure 2. long description.

What constitutes effective interdisciplinary research depends on context and perspective (Budtz Pedersen, 2016). A necessary condition, however, is shared interest in the interdisciplinary research question, which is more likely when research questions are developed collaboratively (Christie, 2011). Engagement may also be easier to sustain when each participating discipline can make a meaningful intellectual contribution (Budtz Pedersen, 2016). The bridge model illustrates three scenarios that differ in the intellectual contribution each researcher can make to the shared research space, represented as a bridge (Figure 2).

In the first scenario, opportunities for meaningful intellectual contribution are unevenly distributed among the researchers. This may occur when the research question is framed primarily through one disciplinary worldview or when collaborators are insufficiently familiar with one another’s expertise and research interests (Christie, 2011).

In the second scenario, opportunities for meaningful intellectual contribution are limited for both researchers. This may result from insufficient disciplinary depth or from limited shared understanding across the participating disciplines. Examples in coastal research include a coastal engineer asking a political scientist to map coastal policies, or a political scientist asking a coastal engineer to produce a risk map, without involving the collaborator in framing the question or considering whether the task is intellectually engaging and relevant to their research interests.

In the third scenario, both (or all) researchers can fully draw on their expertise and are more likely to be genuinely and sustainably engaged in the joint research question. Reaching this stage of collaboration, whether between individual researchers or within teams, requires time, trust, mutual respect and strong relationships. Examples from a coastal research perspective are joint design of research infrastructure or projects, for example, concerning coastal adaptation pathways.

The bridge model is not static: scenarios one and two may produce useful results and can develop toward scenario three as researchers build shared understanding, particularly in emerging interdisciplinary fields.

Although illustrated with two researchers, the bridge model and the hook-shaped researchers can be extended to larger teams. As the number of participating disciplines increases, coordination and integration become more demanding, a concern examined in the emerging field of the science of team science (Stokols et al., 2008; Hall et al., 2012).

Interdisciplinary teams need time to get to know one another and understand each other’s research interests (Stokols et al., 2008; Hall et al., 2012; Kolesar et al., 2024). This process can be supported by creating opportunities for interaction, for example, by providing funding for interdisciplinary teams to develop research ideas without immediate demands for concrete results. In university settings, collaboration in teaching and supervision can lay the groundwork for future research collaborations, as can institutional seed funding programs (Tumer et al., 2024). Colocation creates opportunities for informal interaction (Rittel and Webber, 1973; Institute of Medicine, 2005), but distributed teams can also collaborate effectively. Writing and coding retreats and joint fieldwork campaigns can complement online interactions and help strengthen relationships and mutual understanding.

Strong, sustained teams may be better able to manage the friction that can arise in interdisciplinary collaboration (Christie, 2011). Such friction can foster intellectual development when it challenges disciplinary paradigms, but this requires mutual respect and psychological safety (MacMynowski, 2007; Hall et al., 2012; Apine et al., 2026). Power differences related to seniority, institutional culture and disciplinary hierarchy can affect these conditions (MacMynowski, 2007). Genuine curiosity about others’ perspectives and ways of knowing, together with a reflexive approach to one’s own knowledge, are central to making such processes constructive.

In such contexts, distributed leadership, in contrast to more authoritative leadership styles, can encourage team members to contribute more actively to research ideas and to take responsibility for their own research interests (helping to avoid scenarios one and two in the bridge model) (Budtz Pedersen, 2016). This is particularly important when developing research proposals, where some disciplines risk being included as ‘add-ons’ or assigned overly simple or unrealistic tasks.

Not all disciplinary combinations will result in productive interdisciplinarity. In some cases, multidisciplinary collaboration may be more appropriate. Reconfiguring or dissolving teams when interdisciplinary collaboration has been explored without success can create space for new, sometimes unexpected constellations, in which interdisciplinary collaboration may develop more naturally (Institute of Medicine, 2005; Stokols et al., 2008).

## Funding, publications and awards for interdisciplinary coastal research

Previous studies have reported difficulties in obtaining funding for interdisciplinary research (Bromham et al., 2016). In contemporary coastal research, however, many programs explicitly encourage or require interdisciplinary collaboration (e.g., Coastlines and People Program of NSF). While this development is positive, the application processes and project structures do not always fully support high-level interdisciplinary collaboration.

The broad nature of EU Horizon calls often requires both geographical and disciplinary diversity, which can distribute collaborators across institutions and countries. Interdisciplinary outcomes can also be difficult to accommodate within predefined deliverables and lump-sum funding schemes. Some projects may therefore benefit from strong local interdisciplinary teams complemented by international disciplinary networks (Budtz Pedersen, 2016). Local teams may also facilitate transdisciplinary engagement with stakeholders and citizens.

Long-term interdisciplinary teams and networks are valuable when responding rapidly to new funding calls. In contrast, rapidly assembled consortia may struggle to formulate research questions that are relevant to all partners. Budtz Pedersen (2016) describes how SSH researchers are often invited after the research questions have been defined and most of the budget allocated. Although such projects may not initially reach scenario three in the bridge model, they can provide a starting point for future collaboration.

Difficulties in publishing interdisciplinary research are also frequently mentioned in the literature. Here too, we observe a positive development, with an increasing number of interdisciplinary journals and initiatives, of which this journal is one example. In our experience, interdisciplinary work in dune ecomorphodynamics has often been easier to motivate, develop into compelling research questions and publish than unidisciplinary work on dunes. This reflects the field’s gradual development from initially unidisciplinary perspectives toward more interdisciplinary approaches.

Still, challenges remain in identifying appropriate reviewers for interdisciplinary manuscripts, and review processes can be longer (Zhang and Wang, 2024). Individual researchers can contribute by accepting such review invitations and by being explicit with editors about which parts of a manuscript they are competent to assess and which parts would benefit from additional disciplinary expertise. Increased interdisciplinary training during doctoral education would also make researchers more comfortable reviewing topics where their expertise is needed but covers only part of the manuscript.

The uncertainty associated with outcomes and publications may discourage early-career researchers from engaging in interdisciplinary work (Deininger et al., 2021; Apine et al., 2026). Although criteria for tenure and promotion vary between institutions, rewarding collaboration and interdisciplinary novelty could stimulate engagement among productive faculty members.

Awards also tend to focus on individual achievements rather than collective efforts. Establishing awards that recognize excellence in interdisciplinary research could provide important role models for this form of innovation. In coastal engineering and science, numerous prestigious awards are given to individual scientists, such as the Coastal Award and the International Coastal Engineering Award.

At the 2023 Coastal Sediments Conference, a new award, the Coastal Achievement Award, was introduced. It was awarded to Mitchell Harley and Mike Kinsela for the development of the transdisciplinary research tool CoastSnap (Harley and Kinsela, 2022). Another example is the C. Peter Magrath and W.K. Kellogg Foundation Community Engagement Scholarship Awards administered to teams by the Association of Public Land-Grant Universities in the United States. Such awards shift recognition from individual prestige toward collective outcomes and societal impact, which may encourage early- and mid-career researchers to engage in interdisciplinary collaboration.

## Conclusion

While coastal engineering has long interacted with multiple disciplines, the scale and societal context of climate change adaptation challenges call for both deeper and broader interdisciplinary collaboration.

In this perspective, we have reflected on conditions that may support interdisciplinary coastal research, illustrated by two visual concepts. The hook-shaped researcher describes the combination of profound disciplinary knowledge and reciprocal learning that supports interdisciplinary collaboration. Meanwhile, the interdisciplinary bridge model underscores that collaboration becomes sustainable and meaningful when the joint research space is relevant, fun and challenging for all involved. Our experiences suggest that time, continuity and strong relationships are central to high-level interdisciplinary work. Despite recent positive developments, these conditions are not always well supported by current funding schemes, publication practices and reward structures.

Based on the literature discussed and our experiences, we suggest that universities provide time, funding and space for interdisciplinary relationships to develop. Funders may support early co-development of research questions and allow flexibility as projects evolve. Research leaders can promote shared problem framing, distributed leadership and reciprocal learning, while journals and professional societies can strengthen the recognition of collaborative and interdisciplinary contributions. We hope that the perspective and concepts presented here will stimulate reflection and discussion on how interdisciplinary coastal research can be better supported and more effectively realized in practice.

## Data Availability

A data availability statement is not applicable to this perspective article.

## Long descriptions

### Figure 2. Long description

A vertical arrow on the far left points upward, labeled Intellectual contribution. To the right are three panels separated by vertical lines.

* Scenario 1: Two researchers stand facing each other. The researcher on the left is positioned higher than the researcher on the right. A suspension bridge is tilted diagonally downward from the left researcher’s hands toward the right researcher’s feet, indicating an unequal contribution.

* Scenario 2: Both researchers stand at the same level. A suspension bridge sits on the ground between them, connecting their feet. This represents a low level of intellectual contribution from both parties.

* Scenario 3: Both researchers stand at the same level. A suspension bridge is held high between them, connecting their hands and shoulders. This represents a high and equal level of intellectual contribution from both parties.

Navigate back to Figure 2..
