# Supplementary material for: Building bridges across disciplines: Perspectives on interdisciplinary coastal research
Source: Camb Prism Coast Futur. 2026 Sep 9;4:e27. doi: 10.1017/cft.2026.10049 (PMC13748880; doi:10.1017/cft.2026.10049)
Supplement: Peer review history

# Reviewer report: Review: Building bridges across disciplines: Perspectives on interdisciplinary coastal research — R0/PR2

**DOI:** 10.1017/cft.2026.10049.pr2

**Authors:** Isabel Richter

**Affiliations:** Norwegian University of Science and Technology, Norway

Review: interdisciplinary research cambr prisms

This is a timely perspective on an important topic. I enjoyed reading it and appreciate the authors' effort to stimulate discussion around interdisciplinary research. The two conceptual models are memorable and have the potential to become useful tools for discussion within interdisciplinary teams.

My main comments are less about the quality of the manuscript than about its positioning. First, it is not entirely clear who the target audience is. At different points the manuscript seems to address coastal engineers, interdisciplinary researchers, universities, funders and journals. Clarifying this would also help sharpen many of the recommendations. Second, I am not fully convinced why this needs to be a paper about coastal research. Most arguments seem broadly applicable to interdisciplinary research across many fields, so it would be good to explain what makes coastal research unique in this context. Finally, I think the novelty could be positioned more clearly. The proposed concepts are intuitive and useful, but the manuscript would benefit from explaining more explicitly how they extend existing work on interdisciplinarity and team science.

Detailed comments

1. Reference: “National Research Council (2014, p. 62).” Which National Research Council is this? Please specify the country.

2. While the definitions of multidisciplinary, interdisciplinary, transdisciplinary research and convergence are clear, it is less clear which of these approaches the paper ultimately advocates. These concepts are used somewhat interchangeably throughout the manuscript, although they represent different forms of collaboration. Since the proposed models build on these concepts, I would recommend clarifying from the beginning which type(s) of collaboration they are intended to support.

3. The introduction could position the paper more clearly within the existing literature, also including social science approaches. There is already a substantial body of work on interdisciplinarity, team science, boundary objects and knowledge integration. It would strengthen the paper to explain more explicitly what gap the proposed concepts fill and why existing frameworks are not sufficient. I also think some relevant literature is missing, including practical frameworks for integrating natural and social sciences (e.g., Richter et al., 2022, Building bridges between natural and social science disciplines, Philosophical Transactions of the Royal Society B).

4. The transition from broad coastal adaptation challenges to a focus on coastal engineering feels rather abrupt. Is coastal engineering the primary target audience, or is it mainly used as an example? Clarifying this would make the scope of the paper much clearer.

5. Before introducing the two conceptual models, I would appreciate a short summary of the main barriers identified in previous research (e.g., communication, epistemological differences, incentives, institutional structures, disciplinary identities). This would provide a stronger rationale for why the new concepts are needed.

6. One aspect I missed throughout the manuscript is a stronger international perspective. The discussion focuses almost entirely on disciplinary diversity, but many interdisciplinary coastal projects also involve collaborations across cultures, research systems and institutional contexts, particularly with partners in the Global South. Successful collaboration in these settings depends not only on bridging disciplines, but also on equitable partnerships, capacity building, local and Indigenous knowledge, and navigating different research cultures. Given the global nature of coastal adaptation, this feels like an important omission.

7. The hook-shaped researcher is a nice and memorable concept. However, I am not yet convinced what it adds beyond existing ideas such as T-shaped researchers, boundary spanning, knowledge brokers or interdisciplinary competencies. Does it mainly illustrate existing ideas, or does it generate new insights or recommendations? Related to this, the figure only illustrates collaboration between two researchers. Most interdisciplinary projects involve larger teams, so it would be useful to discuss whether the model also scales to more complex collaborations.

8. The argument that researchers should sometimes accept different terminologies rather than strive for a shared language is interesting because it contrasts with much of the interdisciplinary literature. Can you elaborate on when this approach is preferable and perhaps provide an example where maintaining different conceptual perspectives actually benefited collaboration?

9. I agree that interdisciplinary collaboration is a trainable skill, but the discussion remains quite general. Some concrete examples of educational practices would make the recommendations better

10. The bridge model is visually appealing, but I am less convinced about its conceptual novelty. At present it mainly illustrates the rather intuitive idea that successful interdisciplinary collaboration requires meaningful contributions from all participating disciplines. Could the authors explain more explicitly what new understanding the model provides and how it could influence the way interdisciplinary projects are designed or evaluated? I also wondered whether the three scenarios are presented a bit too statically. Many successful collaborations evolve from scenario one or two towards scenario three. some concrete examples illustrating each scenario would help

11. The recommendation that interdisciplinary teams often benefit from local collaboration deserves a little more nuance. While co-location certainly facilitates interaction, many successful interdisciplinary projects now operate across countries and continents.

12. The discussion of distributed leadership is valuable but remains somewhat abstract. Some concrete examples would make the recommendations easier to apply.

13. Several arguments in the section on funding are presented quite confidently but seem to rely mainly on the authors' own experiences. For example, the statement that obtaining interdisciplinary funding is no longer a major challenge contrasts with parts of the existing literature.

14. I found the discussion of Horizon Europe interesting, but I am not yet convinced by the recommendation that projects should be organised around local interdisciplinary teams with international disciplinary collaborations. Could the authors explain why this structure would work better than current consortium models, or provide examples where this has been successful?

15. The section on publications and awards is useful, although I think it could present a slightly more balanced picture. Interdisciplinary publishing has become easier in many fields through dedicated journals, interdisciplinary editorial boards and funding initiatives. Likewise, recognition could extend beyond team awards to include societal impact, capacity building and long-term collaborative leadership. I also found the statement that “last-minute applications based on rapidly assembled consortia should be avoided” a bit too …categorical? While long-term collaborations clearly have advantages, successful collaborations can also emerge from newly formed teams

16. I think the conclusion could be strengthened. At the moment it mainly summarizes the two conceptual models. I would instead end with a stronger message about what universities, funders and research institutions could do differently to better support high-quality interdisciplinary research.

# Reviewer report: Review: Building bridges across disciplines: Perspectives on interdisciplinary coastal research — R0/PR3

**DOI:** 10.1017/cft.2026.10049.pr3

This article offers a perspective on interdisciplinary coastal research from a coastal engineering point of view and introduces two new concepts. It is well written and structured, with great examples. However, how the concepts were developed is not explained enough and I have some other major and minor comments.

Major comments:

1. The introduction currently focuses very much just on erosion, but the abstract gives the impression that it is about all climate related coastal hazards. Please either revise the abstract or add some brief examples of flooding and flood schemes.

2. You tend to use interdisciplinary research, interdisciplinary researcher and transdisciplinary research interchangeably. I appreciate that the boundaries are not clear but make sure they are all explained and the difference is made when needed.

3. Why did you choose the complexity of research as a metric for successful interdisciplinary collaboration? Is it just to ‘maintain the interest over a project’ as mentioned on L 216 or does it have implications for outcomes and impact? Could there be any other metrics? This needs more explanation as the concept is based on it but currently does not have a strong grounding. I also think that the visualisation with just two researchers might slightly dismiss the complexity of interdisciplinary research teams that involve many researchers from different disciplines.

4. Overall, I think there could be more reminders of the context which is coastal engineering. It is mentioned at the end of the research aim, has its own section, but then is not mentioned again until the conclusions. While obviously this will be relevant to all coastal research, I think you can highlight the importance for coastal engineering as other disciplines might have more interdisciplinary training offered (ecologists, environmental social scientists).

Specific comments:

Figure 1: It is not very illustrative. It is unclear what the dashed lines and grey shades represent. I don’t think you need it.

L119-120: The word ‘interaction’ hints at interdisciplinarity. I think you do not need to mention uni- and multi-disciplinary research at this point anymore but provide a stronger explanation of why understanding and improving interdisciplinary research practices is important.

L126: How does transdisciplinary research fit here if you mention just above that it is about achieving interdisciplinary research?

L167: A reference for the T-shaped researcher would be good or at least an explanation that it is a common concept as opposed to your proposed hook-shaped researcher model.

Figure 2: Similar to Figure 1, it is unclear what the dashed lines and grey shades represent. Perhaps you could visualise the space you mention in the explanation on Lines 172-173 to make it more illustrative.

L170: Is it about individual interdisciplinarity or about the skills to work in an interdisciplinary research team?

L293: Some references would be good.

# Editor report: Recommendation: Building bridges across disciplines: Perspectives on interdisciplinary coastal research — R0/PR4

**DOI:** 10.1017/cft.2026.10049.pr4

**Affiliations:** MaREI, University College Cork, Ireland

Dear Authors

You will see that both reviewers (although providing a different outcome) have made significant and substantive comments regarding revisions that they recommend are made before the manuscript is accepted, whilst recognising the merits of the subject and the work reported. Having read the rviews I agree that the changes would lead to a significant improvement and impact of a final paper and I very much hope that you will revise the manuscript along the lines sugested and re-submit.

Kind regards, Martin

# Editor report: Decision: Building bridges across disciplines: Perspectives on interdisciplinary coastal research — R0/PR5

**DOI:** 10.1017/cft.2026.10049.pr5

**Affiliations:** Geography, Cambridge University, United Kingdom of Great Britain and Northern Ireland

No accompanying comment.

# Editor report: Recommendation: Building bridges across disciplines: Perspectives on interdisciplinary coastal research — R1/PR7

**DOI:** 10.1017/cft.2026.10049.pr7

**Affiliations:** MaREI, University College Cork, Ireland

Dear Authors

Thank you for comprehensively considering the reviewers comments and I consider that all have been satisfactorily addressed. Although there are some questions that remain open I think this is inevitable and you have put forward a perspective based on your research that encourages further debate and makes an important contribution to this area of multi-disciplinary work. I am happy to recommend the manuscript is accepted and look forward to seeing it published.

Regards, Martin

# Editor report: Decision: Building bridges across disciplines: Perspectives on interdisciplinary coastal research — R1/PR8

**DOI:** 10.1017/cft.2026.10049.pr8

**Affiliations:** Geography, Cambridge University, United Kingdom of Great Britain and Northern Ireland

No accompanying comment.
